# ^89^Zr-pro-MMP-9 F(ab′)_2_ detects colitis induced intestinal and kidney fibrosis

**DOI:** 10.1038/s41598-020-77390-7

**Published:** 2020-11-23

**Authors:** Nicole Dmochowska, William Tieu, Marianne D. Keller, Courtney A. Hollis, Melissa A. Campaniello, Chris Mavrangelos, Prab Takhar, Patrick A. Hughes

**Affiliations:** 1 Centre for Nutrition and Gastrointestinal Diseases, Adelaide Medical School, Level 7, University of Adelaide and South Australian Health and Medical Research Institute, Adelaide, South Australia 5000 Australia; 2 Molecular Imaging and Therapy Research Unit (MITRU), South Australian Health and Medical Research Institute, Adelaide, Australia; 3 Preclinical, Imaging and Research Laboratories (PIRL), South Australian Health and Medical Research Institute, Adelaide, Australia

**Keywords:** Molecular medicine, Gastrointestinal models, Inflammatory bowel disease

## Abstract

Intestinal fibrosis is a common complication of inflammatory bowel disease but remains difficult to detect. Matrix metalloproteases (MMPs) have key roles in fibrosis and are therefore potential targets for fibrosis detection. We determined whether immunoPET of F(ab′)_2_ antibody fragments targeting MMPs detects colitis induced colonic fibrosis. Mice were administered 2% dextran sulfate sodium treated water for 1 cycle (inflamed) or 3 cycles (fibrotic), or were untreated (control). Colonic and kidney collagen, innate cytokine, MMPs and fecal MPO concentrations were analyzed by multiplex/ELISA. α-pro-MMP-9 F(ab′)_2_ fragments were engineered and conjugated to ^89^Zr for PET imaging, ex-vivo Cherenkov analysis and bio-distribution. Colonic innate cytokine concentrations and fecal myeloperoxidase were increased in inflamed mice but not fibrotic mice, while collagen concentrations were increased in fibrotic mice. MMPs were increased in inflamed mice, but only pro-MMP-9 remained increased in fibrotic mice. ^89^Zr-pro-MMP-9 F(ab′)_2_ uptake was increased in the intestine but also in the kidney of fibrotic mice, where collagen and pro-MMP-9 concentrations were increased. ^89^Zr-pro-MMP-9 F(ab′)_2_ detects colitis induced intestinal fibrosis and associated kidney fibrosis.

## Introduction

Inflammatory bowel disease (IBD), incorporating Crohn’s disease and ulcerative colitis, is characterised by remitting and relapsing inflammation of the lower gastrointestinal (GI) tract^1^. Intestinal fibrosis is one of the most common IBD related complications, with severe fibrosis leading to stricture and stenosis in ~ 30% of IBD patients^2–4^. Intestinal fibrosis is not currently treatable and can become life threatening, leaving endoscopic mechanical manipulation or surgical resection as the only options^5,6^. Importantly, no validated diagnostic tools or biomarkers currently exist for detecting or staging intestinal fibrosis^5,6^.

Fibrosis is characterised by the excessive deposition of extracellular matrix (ECM) proteins including collagen by persistently activated fibroblasts^7–9^. Fibrosis typically occurs in a progressive manner following repeated episodes of inflammatory damage and remission, or healing, such as occurs in IBD and in the dextran sodium sulphate (DSS) pre-clinical model of intestinal fibrosis^8,10,11^. Matrix metalloproteinases (MMPs) regulate fibrosis by degrading the ECM that is normally laid down as the tissue renews^7,12^. MMP function is tightly regulated by tissue inhibitors of matrix metalloproteinases (TIMPs), which inhibit MMP activity in a 1:1 ratio. MMP-2, -3, -8 and -9, and TIMP-1 are altered to varying extents in fibrotic tissue resected from IBD patients and in pre-clinical models of intestinal fibrosis^10,11,13–15^. However, MMPs and TIMPs are also increased in inflamed intestinal tissue and it remains unclear how their expression is altered in inflamed relative to fibrotic tissue.

Diagnostic tools are ideally sensitive, quantitative and selective for their pathology, non-invasive to the patient, provide information rapidly and do not influence the disease process^5,16^. Stricture can be detected by endoscopy, however endoscopy is limited to imaging only the mucosal surface and not the deeper layers of the colon wall where fibrosis develops^17^. Molecular imaging approaches including positron emission tomography (PET) have superior sensitivity and provide quantitative information^16,18^. ImmunoPET combines the superior target selectivity provided by antibodies with the sensitivity of PET and we, amongst others, have previously demonstrated that immunoPET strategies effectively detect intestinal inflammation^16,19–21^. However, the use of intact antibodies as molecular imaging probes is challenging as their relatively high molecular weight results in long half-lives, potentially translating into increased patient exposure to ionizing radiation, and it may also impede the penetration of fibrotic tissue^22^. Engineering F(ab′)_2_ antibody fragments from intact antibodies by cleaving the effector F_C_ region retains the target specificity of the antibody whilst reducing its molecular weight, thereby increasing excretion and promoting tissue penetration.

Here we aimed to undertake the first immunoPET study of fibrosis in any tissue, pre-clinical or clinical, to determine whether immunoPET of F(ab′)_2_ antibody fragments targeting MMPs can detect DSS colitis induced colonic fibrosis.

## Results

### Repeated cycles of DSS induces colonic fibrosis

**Figure 1:**
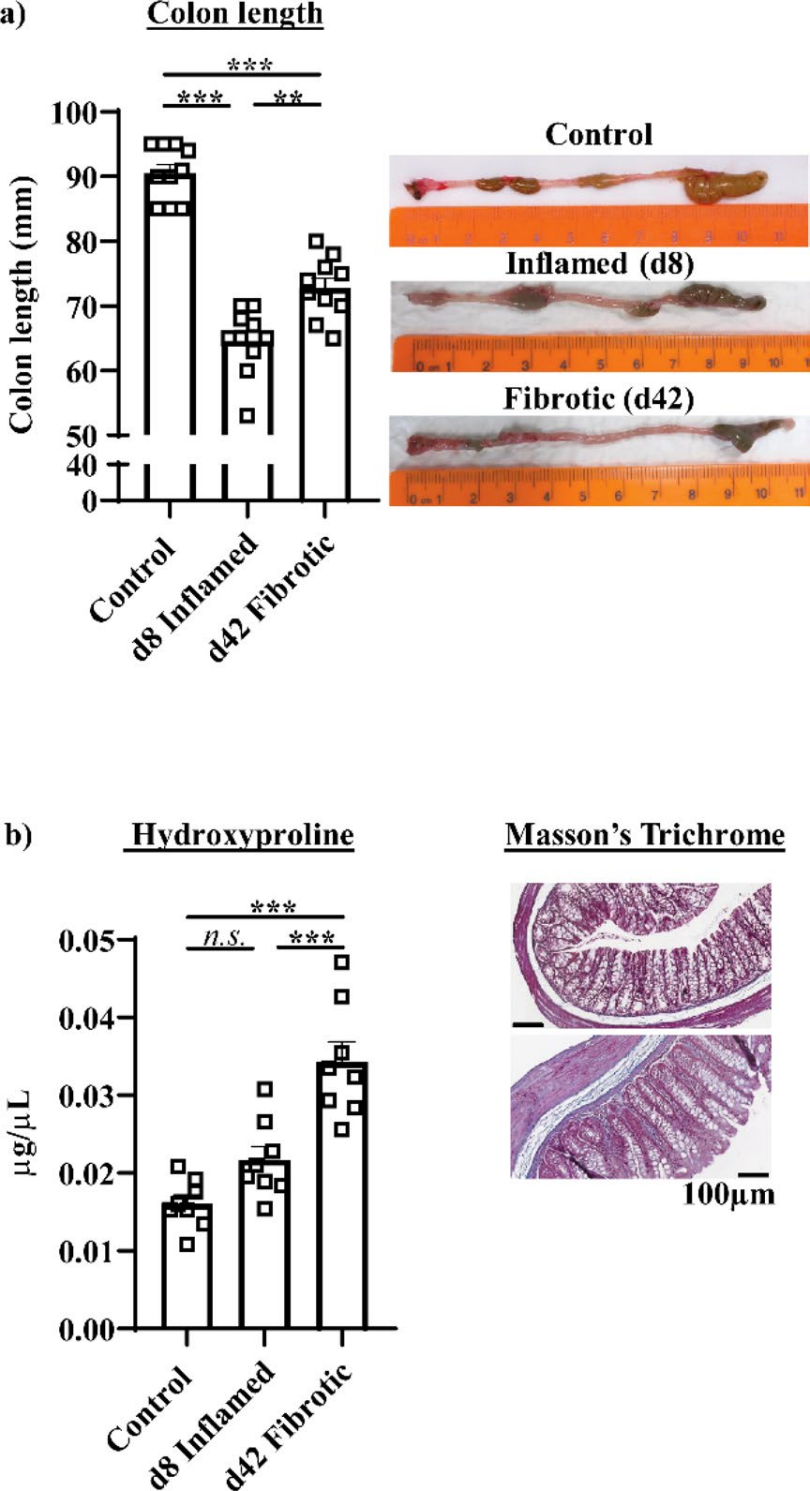
Colonic collagen content increases in inflamed (1 DSS cycle) and fibrotic (3 DSS cycles) mice. (**a**) Colon length did not shorten to the same extent in fibrotic mice as in inflamed mice. (**b**) Repeated DSS cycles increased the colonic collagen content as indicated by hydroxyproline concentration (left) and Masson’s trichrome staining (right) **P < 0.01, ***P < 0.001.

Colon length was significantly (*P* < 0.001) reduced in d8 inflamed mice, however colonic shortening did not occur in fibrotic mice to the same extent as inflamed mice (*P* < 0.01) (Fig. 1a). Collagen deposition was significantly increased in the colons of fibrotic mice relative to inflamed mice and controls as indicated by hydroxyproline content (*P* < 0.001 both) and Masson’s trichrome staining (Fig. 1b).

### Innate cytokines are not altered in fibrotic mice

**Figure 2:**
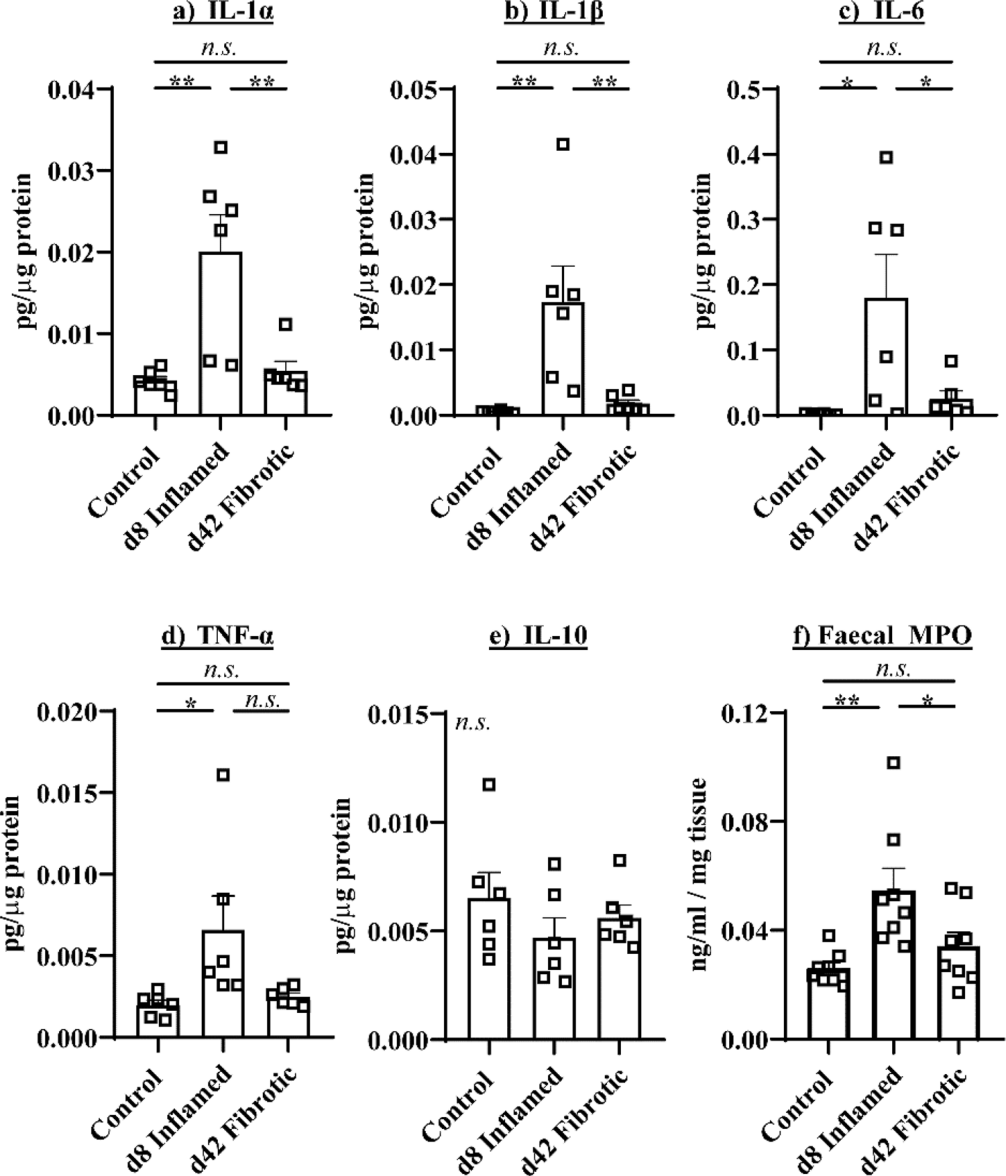
Innate immune mediators are not altered in colonic fibrosis. (**a**) Colonic concentrations of (**a**) IL-1α, (**b**) IL-1β, (**c**) IL-6 and (**d**) TNF-α were increased in inflamed but not fibrotic colons, as are (**f**) fecal MPO concentrations, while (**e**) IL-10 concentrations were not altered in inflamed or fibrotic mice. *P < 0.05, **P < 0.01.

The colonic concentrations of innate cytokines IL-1α (Fig. 2a), IL-1β (Fig. 2b), IL-6 (Fig. 2c) and TNF-α (Fig. 2d), and fecal MPO (Fig. 2f) were significantly increased in inflamed mice relative to controls (*P* < 0.01 IL-1α, IL-1β, faecal MPO. *P* < 0.05 IL-6, TNF-α). However, the colonic concentrations of IL-1α, IL-1β, IL-6 and TNF-α and fecal MPO did not differ between controls and fibrotic mice. IL-10 intestinal concentrations did not differ between d8 inflamed, d42 fibrotic and control mice (Fig. 2e). These results indicate that the key innate immune mediators of intestinal inflammation are not altered in the fibrotic colon.

### Pro-MMP9 remains increased in the fibrotic colon

**Figure 3:**
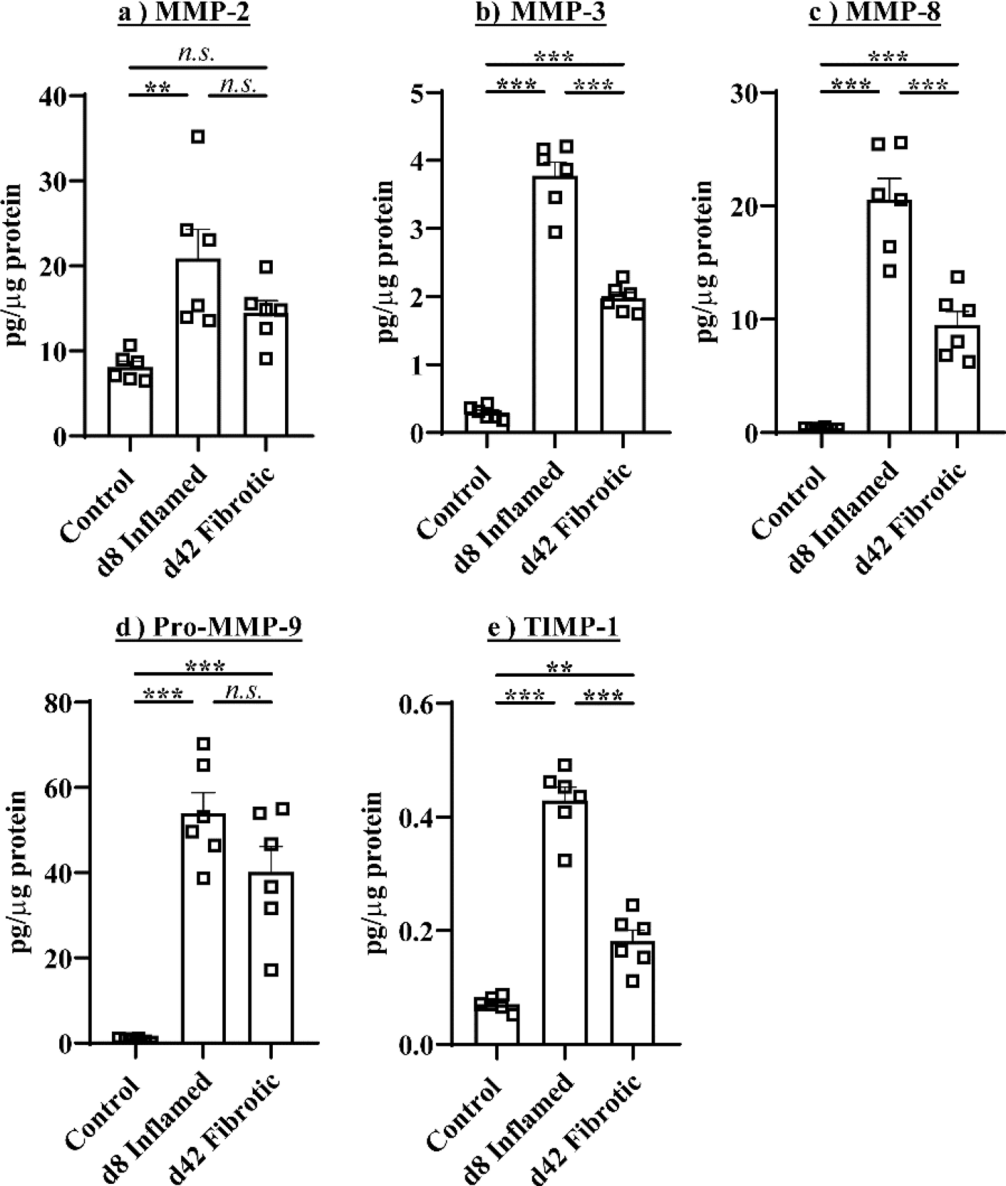
Pro-MMP-9 concentrations remain increased in fibrosis. (**a**) MMP-2, (**b**) MMP-3, (**c**) MMP-8, (**d**) pro-MMP-9 and (**e**) TIMP-1 were increased in inflamed and fibrotic mice, but only pro-MMP-9 remained as high in fibrotic colons as in inflamed colons. *P < 0.05, **P < 0.01, ***P < 0.001.

The colonic concentrations of the MMP members MMP-2 (Fig. 3a), MMP-3 (Fig. 3b), MMP-8 (Fig. 3c), Pro-MMP-9 (Fig. 3d) and TIMP-1 (Fig. 3e) were significantly increased in inflamed mice relative to control mice (*P* < 0.001 MMP-3, MMP-8, pro-MMP-9, TIMP-1, *P* < 0.01 MMP-2). However, while the colonic concentrations of MMP-3, MMP-8 and TIMP-1 were significantly (*P* < 0.001) higher in fibrotic mice than controls, they were substantially reduced compared to inflamed mice. MMP-2 concentrations did not differ in d42 fibrotic mice relative to control mice. Importantly, only pro-MMP-9 concentrations remained as high in fibrotic colons as in inflamed colons.

### ^89^Zr-Pro-MMP-9 F(ab′)_2_ immuno-PET Detects Intestinal fibrosis

**Figure 4:**
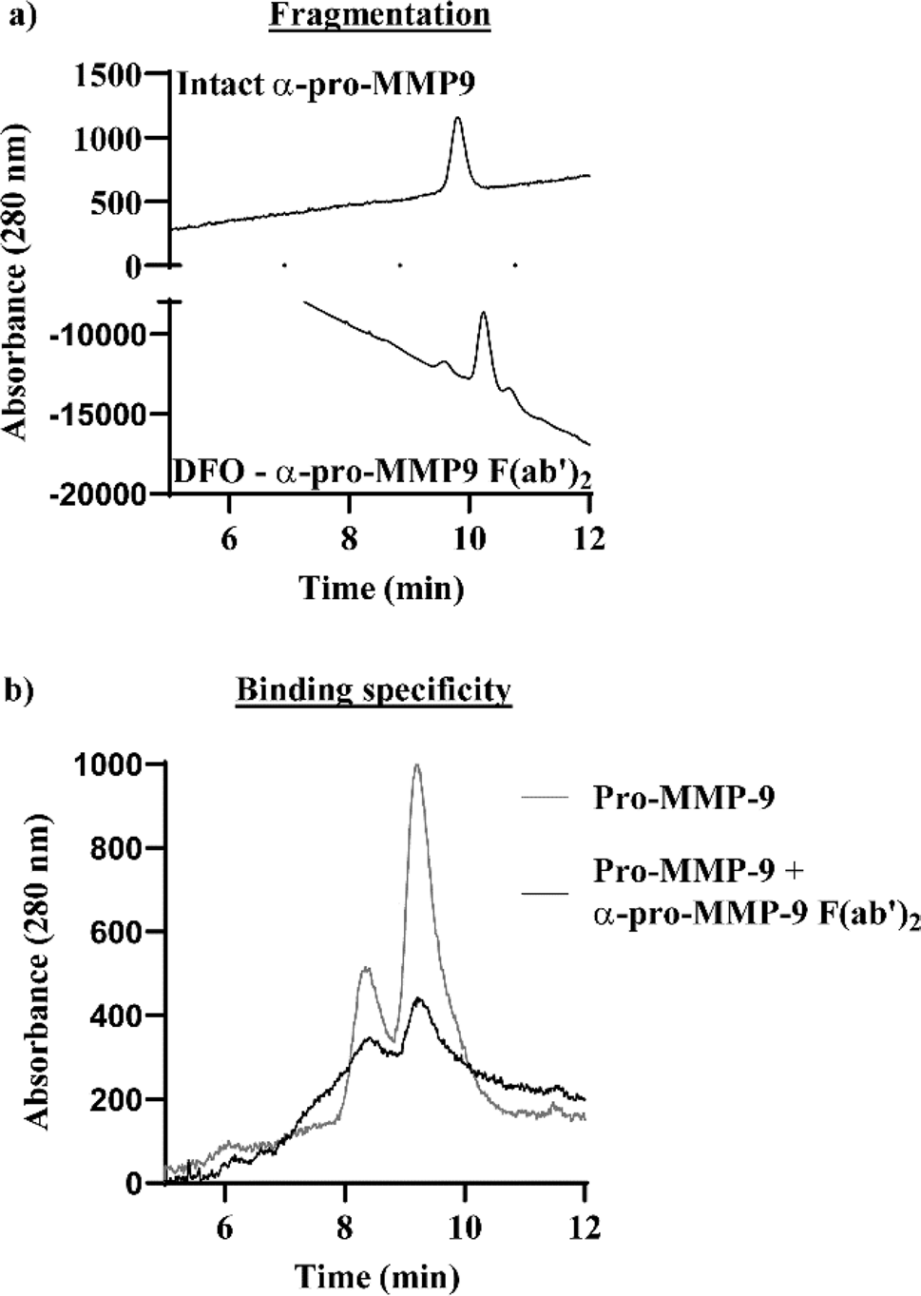
(**a**) α-pro-MMP-9 F(ab′)_2_ fragments had a longer retention time than intact antibody and (**b**) specifically bound pro-MMP-9.

α-pro-MMP9 F(ab′)_2_ fragments had a retention time of 10.24 min and a molecular weight of 103 kDa, while the intact pro-MMP-9 antibody had a retention time of 9.82 min. and a molecular weight of 138 kDa (Fig. 4a). The specificity of our tracer was confirmed by the reduction in pro-MMP-9 signal in samples co-incubated with α-pro-MMP9 F(ab′)_2_ (Fig. 4b).

**Figure 5:**
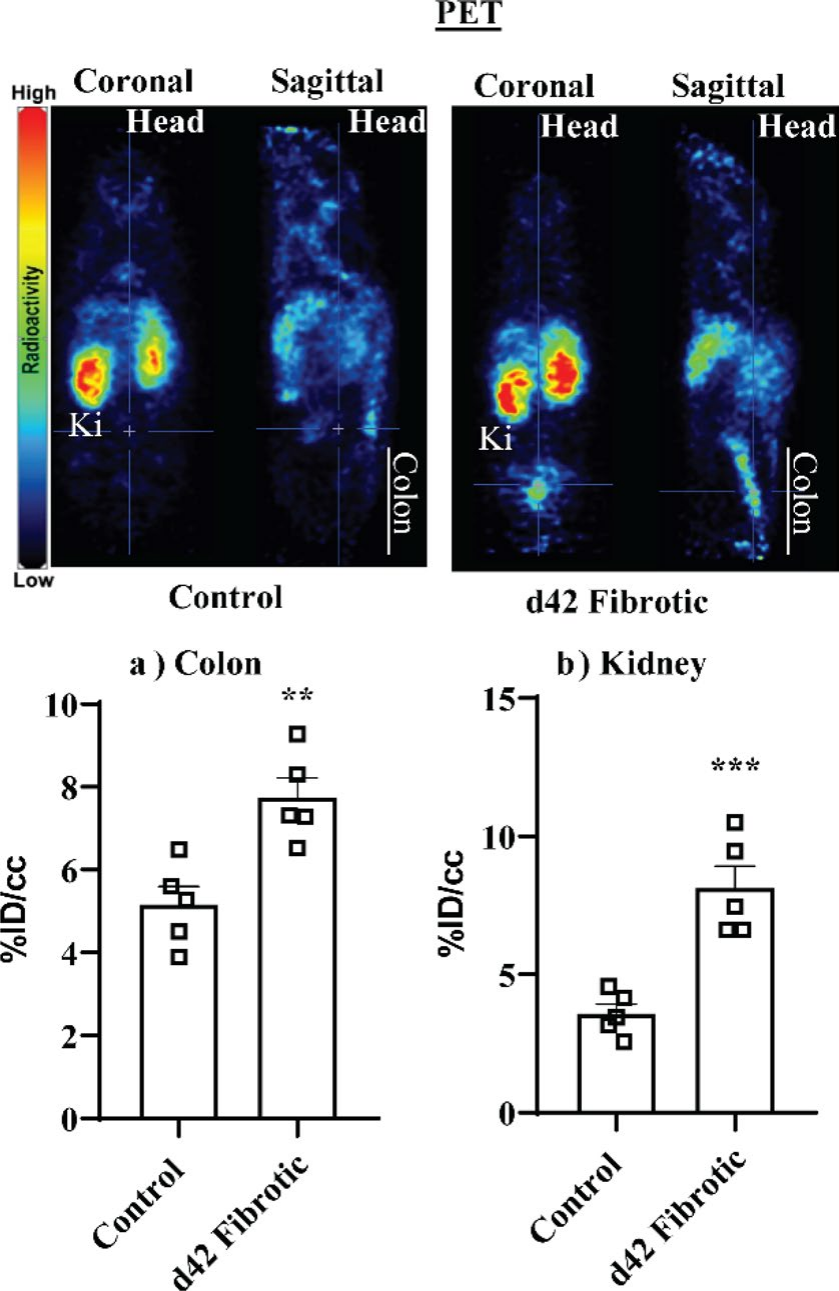
^89^Zr-α-pro-MMP-9 F(ab′)_2_ PET detection of (**a**) colonic and (**b**) kidney fibrosis. Volume of interest (VOI) analysis with representative PET image (coronal and sagittal) of control and fibrotic mice. *Ki* kidney. **P < 0.01, ***P < 0.001.

PET imaging revealed intestinal uptake of ^89^Zr-Pro-MMP-9 F(ab′)_2_ was significantly increased in the colon (*P* < 0.01) (Fig. 5a), but, unexpectedly, also in the kidney (*P* < 0.001) (Fig. 5b) relative to controls [See supplementary video 1 (control) and 2 (fibrotic)].

**Figure 6:**
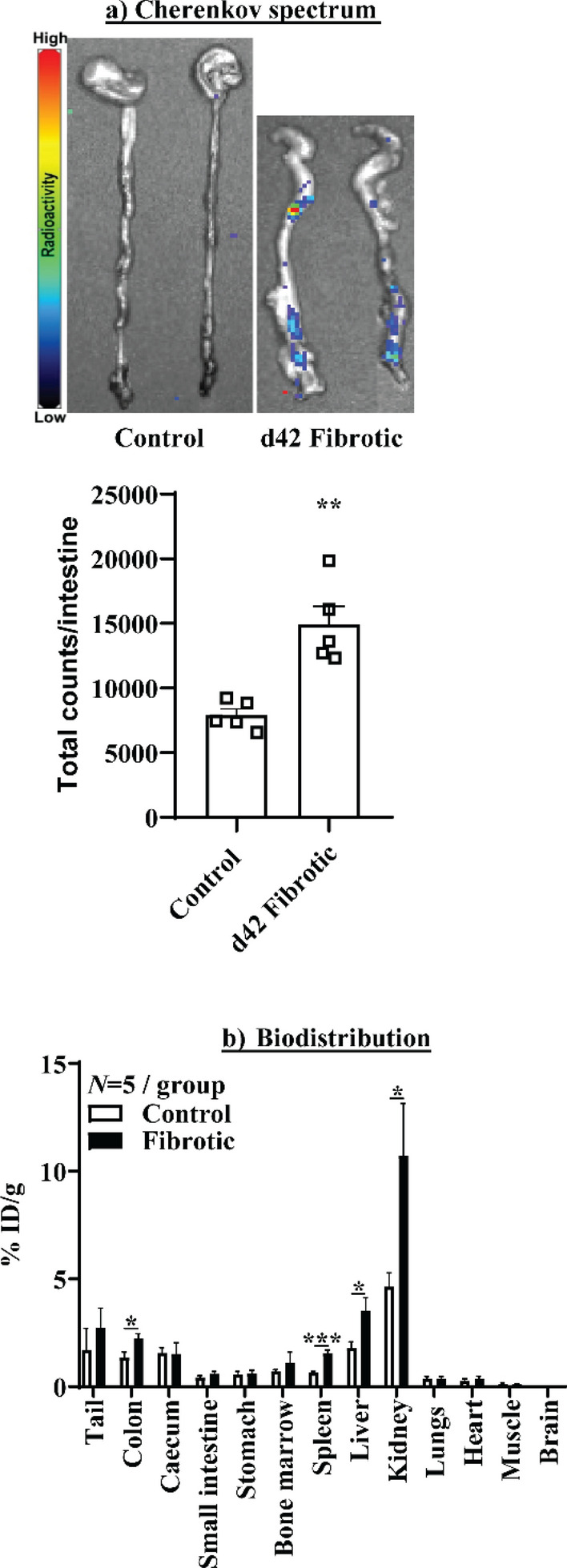
Ex-vivo (**a**) Cherenkov luminescence analysis and (**b**) biodistribution of ^89^Zr-α-Pro-MMP-9 F(ab′)_2_. *P < 0.05, **P < 0.01, ***P < 0.001.

These findings were confirmed by *ex-vivo* analysis that demonstrated that ^89^Zr-α-pro-MMP-9 F(ab′)_2_ uptake was significantly increased in the large intestine (*P* < 0.01) and kidney in d42 fibrotic mice relative to controls by analysis of Cherenkov luminescence (Fig. 6a, *P* < 0.01) and γ-counts (*P* < 0.05 large intestine and kidney, Fig. 6b). ^89^Zr-α-pro-MMP-9 F(ab′)_2_ uptake was not observed in other gastrointestinal sites including the small intestine and caecum, but was significantly increased the spleen and liver (*P* < 0.001 spleen, *P* < 0.05 liver, Fig. 6b). Uptake was similar between DSS treated and control mice in all other tissues investigated.

### Repeated DSS cycles induce kidney fibrosis

**Figure 7:**
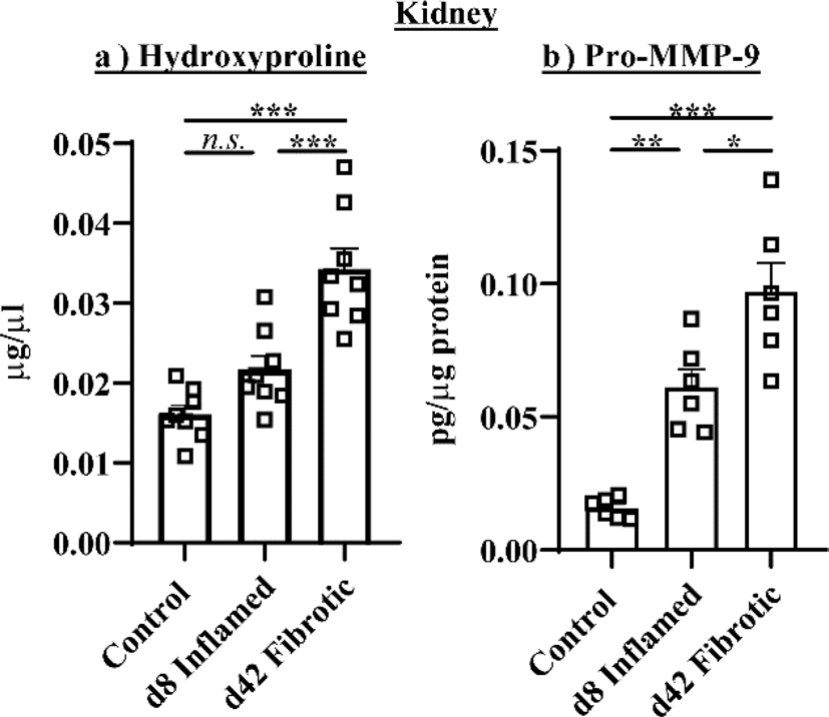
Repeated DSS cycles increased (**a**) hydroxyproline and (**b**) pro-MMP-9 concentrations in the kidney *P < 0.05, **P < 0.01, ***P < 0.001.

Hydroxyproline (Fig. 7a) and pro-MMP-9 (Fig. 7b) concentrations were significantly increased in the kidney of fibrotic mice relative to inflamed mice and controls (*P* < 0.001 hydroxyproline and pro-MMP-9).

## Discussion

Fibrosis is a common and serious complication of IBD but remains difficult to detect and treat. We conducted the first immunoPET study of fibrosis to demonstrate that ^89^Zr labelled F(ab′)_2_ antibody fragments directed against pro-MMP-9 reliably detects colonic fibrosis in the absence of inflammation. We also demonstrate that the pro-MMP-9 signal in the colon co-exists with a strong signal in the kidney, which we also reveal becomes fibrotic in this model. These findings highlight the utility of immunoPET for detecting changes in specific mediators of fibrosis in the organ of interest and also in organs that are distant from the initially affected site.

We compared MMP, TIMP and innate immune cytokine concentrations in inflamed and fibrotic intestines and demonstrate that pro-MMP-9 selectively remains as high in fibrotic tissue as in inflamed tissue. Our findings of increased MMP-2, -3, -9 and TIMP-1 in fibrotic intestinal tissue confirm previous findings in pre-clinical models of intestinal fibrosis^10,11,23^. However MMP and TIMP expression in fibrotic intestinal resections from IBD patients remains controversial; MMP-2, -3, -9 and TIMP-1 are reportedly increased to a similar extent in fibrotic and inflamed intestinal tissue relative to tissue from healthy subjects^14,15^, while others observed that TIMP-1 is increased but MMP-3 is reduced in intestinal fibrosis relative to nearby unstrictured tissue^13^. Furthermore, MMPs and TIMPs are typically also upregulated in inflamed regions of IBD patients, and some have suggested that serum MMP concentrations may be suitable as biomarkers of intestinal inflammation^14,24–28^. These observations highlight the difficulty in identifying unique markers of fibrosis as inflammation and fibrosis can co-exist^6^, highlighting the importance of comparing inflamed and fibrotic tissue. We found that of all the MMPs and innate cytokines investigated, only pro-MMP-9 remained as high in fibrotic tissue as in inflamed tissue indicating that pro-MMP-9 concentrations remain high in the absence of inflammation. Our findings related to MPO are particularly important as MPO is closely related to calprotectin in humans and fecal calprotectin content is a clinically useful marker of intestinal inflammation. Therefore our study demonstrates that high pro-MMP-9 is a biomarker of intestinal fibrosis in the absence of inflammation, and would potentially be of use in IBD patients experiencing symptoms of fibrosis that are concomitant with low faecal calprotectin levels.

There are no validated diagnostic tests for intestinal fibrosis, highlighting a major unmet need as early intervention is prevented. Furthermore, there are no endorsed end-points for clinical trials of urgently needed therapeutics. Endoscopy has long been the backbone tool for diagnosis of intestinal disease, but is only useful for detecting fibrosis in its later stages when stricture and stenosis manifest, and the jejunum and proximal ileum are normally difficult to access by endoscopy^17,29^. Furthermore, there is no accepted histopathological scoring system for grading the severity of intestinal fibrosis in biopsies obtained from endoscopy. Recent developments in MRI, CT and intestinal ultrasound have provided some promise for imaging intestinal fibrosis^5^, however their sensitivity is typically lower than PET and generating quantifiable data can be difficult^18^. Additionally, these technologies typically focus on discrete body regions and therefore potentially miss concurrent disease in other organs, as we observed in the kidney in the current study. Only one study to date has assessed PET/SPECT approaches for detecting fibrosis in IBD patients, demonstrating that PET combined with MR enterography could differentiate fibrotic strictures from inflammation while PET/CT did not perform as well^17,30^. However, the metabolic marker ^18^F-FDG was used as the probe in this study and discriminating the changes in metabolism that are induced in fibrosis from those that are related to inflammation involved changes is likely to be difficult. Advances in the molecular imaging of fibrosis have been made in other tissues, and clinical/pre-clinical studies have now identified useful probes targeting the stomatostatin receptor, ECM constituents including collagen and fibrin, cathepsins and integrins involved in TGF-β activation^18^. However, these are yet to be applied to intestinal fibrosis in clinical or pre-clinical studies, and, to the best of our knowledge, our study is the first immunoPET based approach to identify fibrosis in any tissue.

ImmunoPET directed against immune cells or mediators has demonstrated efficacy for detecting colonic inflammation in pre-clinical models^16,19–21^. We recently demonstrated that targeting inflammation induced immune mediators such as IL-1β results in a substantially more localized bio-distribution than targeting immune cells, presumably as while immune cells migrate to inflamed sites they are also widely distributed throughout the body^20^. These findings prompted our examination of inducible mediators of fibrosis as opposed to markers on the surface of fibroblasts or constitutively secreted mediators such as TGF-β1. Here, using the same model of colonic inflammation as our previous study, we confirmed that IL-1β is the most favorable innate immune cytokine for detecting intestinal inflammation as it had the highest fold increase. While there was also a substantial increase in colonic IL-6 concentrations during inflammation, targeting IL-6 for imaging is complicated by its constitutive secretion from a range of non-immune cell types including muscle, its relatively high serum concentration, and its known variability in human disease states^31^.

Enthusiasm for developing antibody based probes for PET imaging has been tempered by concerns related to the use of ionizing radiation, which are amplified by the high molecular weight of antibodies, and the potential for the probe to interfere with the disease process^5^. Our use of engineered antibody fragments to detect fibrosis extends from studies employing antibody fragments against the T_HELPER_ immune cell surface marker CD4 and the gut homing immune cell integrin β_7_ to detect colonic inflammation in pre-clinical models^19,21^. F(ab′)_2_ antibody fragments have a substantially reduced size compared to intact antibodies which promotes tissue penetration and increases clearance, thereby reducing patient exposure to ionizing radiation^16,22^. Furthermore, F(ab′)_2_ antibody fragments retain target specificity and removal of the effector F_C_ region effectively renders the antibody inert as F_C_ mediated activation of innate immune cells and the complement cascade is eliminated. F(ab′)_2_ fragments may theoretically influence the disease process by neutralizing their target, however this effect is improbable as the antibody dose for their use as probes for immunoPET is much lower than when they are used as conventional drugs^18^. The dose of ionizing radiation we used in our current study was similar to that used in these previous studies and much lower than the dose we previously used with conjugated intact antibodies to detect colonic inflammation^20^, reducing exposure to radiation^19,21^. Furthermore, it should be noted that the ionizing radiation dose for whole body ^18^F-FDG PET scans is much lower than typically used for abdominal pelvic CT scans, but much more information regarding distant organs can be obtained^16,32,33^. Importantly, immunoPET offers the potential for both early detection and theranostic treatment which are critically needed for treatment in the intestine and other fibrotic tissues.

We observed that the increased pro-MMP-9 signal in the fibrotic colon occurred concurrently with increased signal in the kidney, which we demonstrate for the first time develops fibrosis in this model. IBD is frequently associated with extra-intestinal manifestations. Nephritis and nephrolithiasis, or stone formation, is the most frequently experienced renal diseases in IBD patients, and is particularly prevalent in those with long standing disease^34^. MMP-9 expression is increased in nephritis and nephrolithiasis has been linked to MMP-9 polymorphisms, however in relation to IBD it remains difficult to determine whether these diseases result from immune mechanisms that are shared or distinct to those in the intestine, or whether they result from drug toxicity^34,35^. Kidney injury and increased inflammatory mediator content has previously been demonstrated in DSS induced colitis^36^. However, our study is the first to investigate the kidney in a model of intestinal fibrosis. The mechanisms underlying colitis induced kidney fibrosis that we observed warrant further study.

## Conclusions

We performed the first immunoPET study in any organ, clinical or pre-clinical, and demonstrated that pro-MMP-9 F(ab′)_2_ fragments detected inflammation induced intestinal fibrosis after inflammation had resolved. Furthermore, we also established the kidney becomes fibrotic in this model, underscoring the benefit of PET for detecting disease in organs that are distant from the original disease site and are therefore potentially ignored. As the mechanisms underlying fibrosis are broadly similar across all organs, and ~ 45% of all natural deaths in the western world can be attributed to fibroproliferative disease^8^, we propose that immunoPET of pro-MMP-9 antibody fragments are a valuable addition to the detection of fibrosis in all tissues.

## Methods

All experiments were approved by the Animal Ethics Committee of the South Australian Health and Medical Research Institute (SAHMRI) and The University of Adelaide. All experiments were performed in accordance with the guidelines and regulations of the Animal Welfare Act 1985 (South Australia) and the NHMRC Code for the care and use of animals for scientific purposes (2013, Australia).

### Mice

Male C57BL/6jSah mice aged 10–14 weeks (20–30 g) were bred and group housed in a specific pathogen-free environment at the South Australian Health and Medical Research Institute (SAHMRI). Animals had ad libitum access to food and water. Experiments were conducted with only male mice to eliminate the potential confounding effects of the estrus cycle. Mice were humanely euthanized via CO_2_ inhalation and cervical dislocation to remove tissues.

### DSS colitis model

Colonic inflammation was induced by the addition of 2% (w/v) dextran sodium sulphate (DSS) (molecular mass 40–50 kDa, Alfa Aesar, Lancashire, United Kingdom) for 5 days followed by an additional 3 days of normal drinking water for inflamed mice (d8 inflamed)^20^, while fibrosis was induced by repeating 3 cycles of 5 days DSS treatment followed by 9 days of normal drinking water. Mice were assessed daily for signs of colitis including body weight and diarrhea. Healthy mice were age and weight matched. Colon length was measured from the tip of the anus to the distal end of the colon.

### Hydroxyproline assay

Hydroxyproline concentrations were determined essentially as described in the manufacturer’s protocol (Abcam, Cambridge, UK). Briefly, ~ 1 cm sections of distal colon or kidney were excised free of surrounding fat and blood vessels and homogenized in 100 µL dH_2_O/10 mg tissue before being hydrolyzed in 10 N NaOH (Sigma, Sydney, Australia) for 1 h. at 120 °C, then neutralized with 10 N HCl (Chem Supply, Gillman, Australia) and the supernatant collected after centrifugation. Samples and standards were evaporated on a 96 well flat bottom plate, oxidized (Chloramine-T for 20 min), and absorbance measured at 560 nm after 4-(dimethylamino)benzaldehyde incubation for 45 min at 65 °C. Hydroxyproline concentrations were determined based on their relationship with standards of known concentration and normalized to tissue weight.

### Histology

1 cm sections of distal colon were excised with surrounding fat, stored in 4% formalin at 4 °C and mounted in paraffin blocks. 10 × 4 µm sections of formalin fixed (4 °C overnight) paraffin embedded sections were stained with Masson’s Trichome and processed for image capture [Nanozoomer (Hamamatsu, Japan)].

### Innate cytokine and matrix metalloprotease concentrations

Proteins were extracted from a 1 cm section of distal colon or kidney as previously described^20,37–39^. Concentrations of the innate cytokines IL-1α, IL-1β, IL-6, IL-10 and TNF-α (Merck Millipore, Massachusetts, USA) and matrix metalloproteases (MMP) MMP-2, MMP-3, MMP-8 and Pro-MMP9 (Merck Millipore) were determined by multiplex assay, while TIMP-1 concentrations were determined by ELISA (R&D systems, Minnesota, USA). Kidney pro-MMP-9 concentrations were determined by ELISA (R&D systems). Concentrations were normalized to total protein concentration as determined by a BCA assay (Abcam, UK) as previously described^20,38,39^.

### MPO concentrations

Luminal fecal pellets were removed and homogenized for MPO processing and supernatants collected as previously described^20,37^. MPO concentrations were determined by ELISA essentially as per manufacturer’s instructions (ThermoFisher Scientific, Waltham, MA, USA), with MPO concentrations determined based on a relationship to standards of known concentration and results standardized to fecal wet weight. The limit of sensitivity was < 14 ng/mL.

### F(ab′)_2_ generation, conjugation and radiolabeling

Rat α-mouse pro-MMP-9 IgG_2A_ (R&D Systems Clone # 116134) F(ab′)_2_ fragments were generated by digesting on an immobilized pepsin agarose resin for 4 h at 37 °C, followed by purification on a protein A column essentially as per manufacturer’s instructions (ThermoFisher Scientific). Intact and fragmented antibodies were collected and analysed by SEC-HPLC (LC-20, Shimadzu, Kyoto, Japan) with an AdvanceBio SEC 300 column (7.8 × 300 mm, 2.7 µm, Agilent Technologies, Foster City, USA) under isocratic conditions, with peaks detected at 280 nM. Mass was calculated from a 15–600 kDa protein standard mix (Sigma). ^89^Zirconium (^89^Zr) production and antibody radiolabelling and conjugation was performed essentially as previously described^20^. Briefly, ^89^Zr-oxalate was produced via proton irradiation of an ^89^Y target on a PETtrace 880 cyclotron (GE Healthcare, Illinois, USA) and purified on the ALECO solid target processing system (Comecer, Italy). NCS-Bz-DFO (Macrocyclics, Texas, USA) was conjugated to α-pro-MMP-9 F(ab′)_2_ fragments and radiolabelled with ^89^Zr. Purity was confirmed by SEC-HPLC as described above. Specificity was determined by shift assay (SEC-HPLC) following incubation of recombinant pro-MMP-9 (R&D systems, 5 mg/mL) in the presence or absence of α-pro-MMP9 F(ab′)_2_ in 1:10 ratio for 2 h at room temp.

### MRI-PET

1–2 MBq of ^89^Zr-α-pro-MMP-9 F(ab′)_2_ (71 µg) in 100–200 µL of normal saline was administered intravenously (tail vein) 18 h. prior to imaging and PET scans performed 18 h later over 30 min using a submillimeteric-resolution (0.7 mm) (Albira PET-SPECT small animal scanner, Bruker Biospin GmbH) as previously described^20^. Manually drawn volumes of interest (VOI) were analyzed as previously described^20^.

### Ex-vivo analysis

Organs were dissected immediately after PET imaging, weighed and intestinal ^89^Zr accumulation counted by Cherenkov luminescence imaging performed with an optimal imager (IVIS Lumina, Perkin Elmer USA) equipped with a CCD camera (exposure time 600 s, binning factor 8, field of view 23 cm, in open filter mode), with counts background corrected. ^89^Zr biodistribution was determined in organs using a Hidex γ-counter (Hidex, Finland) with counts background and decay-corrected as previously described^20^.

### Statistical analysis

Data are expressed as mean ± SEM in all cases. All data was determined by Shapiro Wilk testing to have normalized distribution. The significance of results were determined by two-tailed unpaired *t* test or one-way ANOVA with Tukey’s post-hoc test. Differences with *P* < 0.05 were considered statistically significant.

## Supplementary information

Supplementary Video 1.

Supplementary Video 2.

Supplementary information.

## Data Availability

All data generated or analysed during this study are included in this published article and its Supplementary Information files.
