# Supplementary material for: 89Zr-pro-MMP-9 F(ab′)2 detects colitis induced intestinal and kidney fibrosis
Source: Sci Rep. 2020 Nov 23;10:20372. doi: 10.1038/s41598-020-77390-7 (PMC7683569; doi:10.1038/s41598-020-77390-7)
Supplement: Supplementary file 3 — Supplementary information. [file 41598_2020_77390_MOESM3_ESM.pdf]

	multiplex results (pg/mL)					BCA total protein (µg/mL)	converted pg/µg/mL				
	IL-1α	IL-1β	IL-6	TNF-α	IL-10		IL-1α	IL-1β	IL-6	TNF-α	IL-10
Control 1	23.93	1.65	2.96	6.85	42.66	6337.87	0.003776	0.00026	0.000467	0.001080805	0.006731
Control 2	27.82	3.07	13.67	6.56	62.21	5304.844	0.005244	0.000579	0.002577	0.001236606	0.011727
Control 3	19.08	2.46	10.92	15.8	34.02	7743.463	0.002464	0.000318	0.00141	0.002040431	0.0043934
Control 4	7.69	4.48	14.43	13.15	29.26	5610.34	0.001371	0.000799	0.002572	0.002343886	0.0052154
Control 5	23.93	3.12	10.92	14.81	46.01	6333.636	0.003778	0.000493	0.001724	0.002338309	0.0072644
Control 6	18.61	1.79	15.6	13.49	22.29	4572.411	0.00407	0.000391	0.003412	0.002950303	0.0048749
Day 8 inflamed 1	90.72	76.08	1136	34.02	32.35	4005.093	0.022651	0.018996	0.283639	0.008494184	0.0080772
Day 8 inflamed 2	68.8	65.42	254.38	180.02	30	11185.47	0.006151	0.005849	0.022742	0.01609409	0.0026821
Day 8 inflamed 3	32.35	18.14	431.34	15.6	32.35	4847.603	0.006673	0.003742	0.08898	0.003218086	0.0066734
Day 8 inflamed 4	90.72	150.01	6.47	16.83	10.3	3611.358	0.025121	0.041538	0.001792	0.004660297	0.0028521
Day 8 inflamed 5	260.85	146.29	2278	25.48	27.82	7942.67	0.032842	0.018418	0.286805	0.003207989	0.0035026
Day 8 inflamed 6	210.65	122.67	3100	31.55	34.89	7851.301	0.02683	0.015624	0.394839	0.004018442	0.0044438
Day 42 fibrotic 1	28.53	24.53	74.19	13.3	30.77	6348.783	0.004494	0.003864	0.011686	0.00209489	0.0048466
Day 42 fibrotic 2	27.13	5.2	70.55	16.41	30	5493.183	0.004939	0.000947	0.012843	0.002987339	0.0054613
Day 42 fibrotic 3	60.67	16.2	453.59	17.69	44.86	5443.48	0.011145	0.002976	0.083327	0.003249759	0.0082411
Day 42 fibrotic 4	21.1	5.95	178.89	10.92	27.13	5738.147	0.003677	0.001037	0.031176	0.001903053	0.004728
Day 42 fibrotic 5	22.75	5.63	22.75	13.3	30.77	5045.857	0.004509	0.001116	0.004509	0.002635826	0.0060981
Day 42 fibrotic 6	19.08	4	39.56	11.24	22.19	5230.468	0.003648	0.000765	0.007563	0.002148947	0.0042425

	MMP2	MMP3	MMP8	Pro-MMP-9		MMP2	MMP3	MMP8	Pro-MMP-9
Control 1	47880	1237	1632	5313	5304.844	9.025714	0.233183	0.307643	1.001537521
Control 2	39400	2514	2026	5731	5841.103	6.745301	0.430398	0.346852	0.981150253
Control 3	28470	1122	1562	2774	5997.312	4.747126	0.187084	0.26045	0.462540522
Control 4	48929	2057	2210	5670	5610.34	8.721218	0.366644	0.393916	1.01063395
Control 5	41144	1470	2258	1205	6337.87	6.491771	0.231939	0.356271	0.190126969
Control 6	50547	2394	3798	5670	7743.463	6.5277	0.309164	0.490478	0.73223053
Day 8 inflamed 1	103830	18737	114979	173958	4400.6	23.59451	4.257828	26.12803	39.53051857
Day 8 inflamed 2	173958	19104	125649	229430	4940.744	35.20887	3.866624	25.43119	46.43632352
Day 8 inflamed 3	55937	13850	84103	281355	4005.093	13.96647	3.458097	20.99901	70.24929663
Day 8 inflamed 4	48929	15196	59163	235870	3611.358	13.54864	4.207835	16.38248	65.31337936
Day 8 inflamed 5	109051	18081	92527	223406	4687.2	23.2657	3.857527	19.74036	47.6629971
Day 8 inflamed 6	80784	15503	75023	279636	5258.87	15.36148	2.947972	14.26599	53.17416579
Day 42 fibrotic 1	103830	10024	56361	244204	5230.468	19.851	1.916463	10.77552	46.68874865
Day 42 fibrotic 2	69490	12562	34401	173958	5493.183	12.65022	2.286834	6.262489	31.66797757
Day 42 fibrotic 3	80784	11371	74838	299142	5443.48	14.84051	2.088921	13.74819	54.9541813
Day 42 fibrotic 4	93984	11109	43489	109051	6348.783	14.80347	1.749784	6.849974	17.17667838
Day 42 fibrotic 5	45850	8986	40501	185253	5045.857	9.086662	1.780867	8.026585	36.71388113
Day 42 fibrotic 6	89372	11682	64603	309804	5738.147	15.57506	2.035849	11.25851	53.99024832
